# Combining Intra- and Interindividual Approaches in Epistemic Beliefs Research

**DOI:** 10.3389/fpsyg.2020.00570

**Published:** 2020-04-16

**Authors:** Tom Rosman, Eva Seifried, Samuel Merk

**Affiliations:** ^1^Leibniz Institute for Psychology Information (ZPID), Trier, Germany; ^2^Institute of Psychology, Heidelberg University, Heidelberg, Germany; ^3^Institute of Education, University of Tübingen, Tübingen, Germany

**Keywords:** epistemic beliefs, intraindividual differences, interindividual differences, higher education, biology, psychology

## Abstract

We combined inter- and intraindividual approaches to investigate university students’ biology- and psychology-specific specific epistemic beliefs (beliefs about the nature and structure of knowledge). We expected that university students would perceive the discipline of biology as more absolute and less multiplistic than the discipline of psychology (intraindividual perspective). Furthermore, we expected students from so-called “hard” disciplines to perceive biology as more absolute and less multiplistic than students from soft disciplines (interindividual perspective). Finally, we expected that students from hard disciplines, compared to their peers from soft disciplines, would perceive stronger differences between biology and psychology (combined perspective). Hypotheses were tested, using Bayes factors, in *N* = 938 university students from a multitude of disciplines. Results revealed that university students perceive biology as considerably more absolute and less multiplistic compared to psychology. However, the findings also suggest that there are no strong interindividual differences between students from hard and soft disciplines regarding the perception of biology. Finally, results revealed that students enrolled in harder disciplines perceive a slightly stronger difference between biology and psychology. In sum, intraindividual effects were considerably stronger, which elicits doubt that students from hard disciplines espouse a fundamentally different set of epistemic beliefs than their peers from soft disciplines.

## Introduction

Do mathematics students perceive sociological findings as less certain than biological findings? Do psychology students think differently about psychology compared to engineering students? Since the 1970s, questions like these have been investigated under the umbrella term *epistemic beliefs*, defined as individual conceptions about knowledge and knowing (Kuhn and Weinstock, 2002). To date, a vast body of research exists on the relationships of epistemic beliefs with self-regulated learning (e.g., Muis et al., 2015), critical thinking (e.g., Chan et al., 2011), information processing (e.g., Kardash and Howell, 2000), and academic achievement (cf. the meta-analytic review by Greene et al., 2018). Moreover, considerable efforts have been put into investigating whether epistemic beliefs are domain-general or domain-specific, with a general consensus emerging that individuals hold beliefs on different levels (Buehl et al., 2002; Merk et al., 2018).

Empirical studies on the specificity of epistemic beliefs often adopt either inter- or intraindividual perspectives (Muis et al., 2006): Under an interindividual perspective, researchers investigate differences in the epistemic beliefs of two separate populations. In such studies (e.g., Paulsen and Wells, 1998; Karimi, 2014), students from soft disciplines (e.g., psychology, literature, history) often report viewing scientific knowledge as more tentative and less certain than students from hard sciences (e.g., mathematics, physics, biology). Researchers adopting an intraindividual perspective, in contrast, compare the epistemic beliefs of one population with regard to at least two domains or topics. For example, Buehl et al. (2002) contrasted undergraduate students’ beliefs about mathematics with the same students’ beliefs about history. In their study, they found, among others, that mathematical knowledge is perceived as more integrated with other domains than knowledge from the history domain.

While there are several studies adopting either an intra- or an interindividual perspective (see below), previous research has neglected the combination of the two approaches, that is, investigating *interactions* between them. This would allow one to investigate whether different populations systematically differ in their beliefs regarding different domains. For example, since they are socialized differently in terms of the criteria they employ to evaluate evidence, students with a natural science background might perceive psychology as more tentative than biology, compared to students from social science backgrounds.

Identifying such patterns would have considerable value since it allows a better understanding of the dynamics and extent of domain-specific variation in epistemic beliefs. Considering the small but important relationship between epistemic beliefs and academic achievement (Greene et al., 2018), researchers might, for example, come to more exact conclusions on why certain students devaluate certain disciplines, prematurely terminate their studies, or simply fail at their exams. Moreover, higher education is becoming more and more interdisciplinary (e.g., Stoecker, 1993; Borrego and Newswander, 2010), which is why knowledge on how students from different backgrounds perceive different disciplines may help to reduce misconceptions about certain disciplines, and contribute to increasing the attractiveness of interdisciplinary courses.

For these reasons, we conducted a study combining intra- and interindividual approaches. We asked students from a multitude of different disciplines about their epistemic beliefs regarding several disciplines and analyzed the data via Bayesian statistics. Our efforts were led by the following overarching research question: Do students’ discipline-specific epistemic beliefs vary with regard to different disciplines, and to what extent does this variation depend on the students’ own study discipline?

### Theoretical Approach

The present article uses the developmental perspective on epistemic beliefs as theoretical underpinning. Under this perspective, Kuhn and Weinstock (2002) distinguish three types of epistemic beliefs: absolutism, multiplism, and evaluativism. Individuals with high absolute beliefs view knowledge as an accumulation of certain and absolute “facts” or “truths.” Individuals with high multiplistic beliefs, in contrast, stress the subjectivity of knowledge and tend to perceive all viewpoints on a topic as equally legitimate (Hofer and Pintrich, 1997). Finally, individuals endorsing evaluativistic beliefs see themselves as part of the process of knowledge by evaluating and weighing different viewpoints (Kuhn and Weinstock, 2002).

Since the mid-2000s, a general consensus has emerged that epistemic beliefs can be conceptualized on different levels of specificity that interact with each other (Buehl et al., 2002; Muis et al., 2006, 2016). For example, according to the Theory of Integrated Domains in Epistemology (TIDE; Muis et al., 2006), individuals differ in their general epistemic beliefs, their domain-specific epistemic beliefs, and, following the framework’s extension by Merk et al. (2018), in their topic-specific epistemic beliefs. One individual might thus have different beliefs regarding psychology and biology (domain-specific beliefs). However, he or she might also have beliefs regarding academic knowledge in general (academic epistemic beliefs) or regarding specific topics (topic-specific epistemic beliefs; e.g., beliefs regarding the topic of gender stereotyping). Bråten and Strømsø (2010) argue that “personal epistemology at different levels of specificity may have strongest impact on facets of academic learning at comparable levels of specificity” (p. 640). Hence, when investigating how students learn in specific disciplines (e.g., psychology), adopting a domain- respectively discipline-specific^1^ view on epistemic beliefs seems particularly fruitful, especially since it allows making predictions for entire disciplines. For the present research, we therefore adopted a discipline-specific approach to investigate inter- and intraindividual differences—as well as their combination—in how individuals perceive scientific knowledge.

### Intraindividual Perspective: Psychology- vs. Biology-Specific Epistemic Beliefs

Prior research (e.g., Bernstein, 1996; Young and Muller, 2013) has shown that at the higher education level, knowledge structures strongly differ between so-called “hard” and “soft” disciplines. The body of knowledge that is covered in classes from hard disciplines is rather well-structured, draws on more clearly defined concept definitions, and uses more established methodological procedures, so that answers to problems can often be found using formal reasoning and experimentation (Schommer-Aikins et al., 2003; Muis et al., 2006). In contrast, knowledge taught in softer disciplines often exhibits a more ill-defined knowledge structure (Schommer-Aikins et al., 2003; Muis et al., 2006, 2016): Compared to hard disciplines, concept definitions and terminology are often vaguer, methodological procedures are more diverse, and the frequency of inconclusive results is higher (Muis et al., 2006; Karimi, 2014). With respect to these differences in knowledge structures, it is not surprising that many intraindividual studies found that higher education students’ discipline-specific epistemic beliefs differ depending on what discipline they are related to. For example, Muis et al. (2016) found that university students believe that knowledge in mathematics is rather absolute (i.e., fixed/certain), whereas they view knowledge in psychology as more multiplistic (i.e., variable/tentative). Furthermore, Hofer (2000) found that first-year college students view knowledge in psychology as less certain and unchanging than in natural science, and also perceive that, in natural science, experts are more important in attaining the truth than in psychology. Finally, in yet another study, university undergraduates viewed biological knowledge as more certain and trustworthy in contrast to psychological knowledge (Estes et al., 2003), a finding that was later corroborated by Rowley et al. (2008).

To sum up, students generally seem to view harder disciplines as more absolute and less multiplistic than softer disciplines. While some authors claim that such findings reflect that students generally hold more “sophisticated” or “advanced” epistemic beliefs regarding softer disciplines (e.g., Green and Hood, 2013), we think that these differences reflect, to a large extent, differences^2^ in knowledge structures between the respective disciplines. As outlined above, due to the discipline’s more ill-defined knowledge structure (Muis et al., 2006), claims in psychology simply *are* less certain and more tentative in contrast to biology. Hence, the explanation that students simply recognize these differences in knowledge structures, which is then reflected in their discipline-related epistemic beliefs seems more convincing than the idea of an “increased readiness to develop sophisticated [epistemic beliefs] regarding psychology than some other topics” (Green and Hood, 2013, p. 170).

When adopting Biglan’s (1973) classification of disciplines, comparisons between biology and psychology might be particularly interesting. In fact, these disciplines fundamentally differ on one of Biglan’s dimensions since biology is generally considered a hard discipline, whereas psychology is viewed as soft (Biglan, 1973; Stoecker, 1993; Simpson, 2017). On the other hand, both disciplines belong to the “life” domain and are mostly considered as “pure” (Biglan, 1973; Stoecker, 1993; Simpson, 2017). Since Biglan’s (1973) hard/soft dimension is more strongly related to the knowledge structure of a discipline than the two other dimensions (Rosman et al., 2017), differences in epistemic beliefs should be strongest between hard and soft disciplines. To be able to conclusively trace back differences in epistemic beliefs to the hard/soft categorization, we therefore chose two disciplines that only differ on this dimension—at least according to the Biglan scheme.

In an effort to replicate the aforementioned findings on intraindividually varying beliefs regarding hard and soft disciplines, we posit the following hypotheses^3^ :

*Hypothesis 1a:* University students have higher biology-specific absolute beliefs compared to psychology-specific absolute beliefs.
*Hypothesis 1b:* University students have lower biology-specific multiplistic beliefs compared to psychology-specific multiplistic beliefs.

### Interindividual Perspective: Differences in Epistemic Beliefs Between Students From Hard and Soft Disciplines

While a discipline’s knowledge structure surely is as a central predictor of students’ discipline-specific epistemic beliefs, interindividual differences are also important. In fact, students might deliberately choose a certain field of study that conforms with their general (i.e., domain-unspecific) epistemic beliefs because, for example, “students with strong beliefs in the certainty of knowledge may find fields that seem to be characterized by ‘absolute,’ rather than tentative, knowledge to be more attractive” (Trautwein and Lüdtke, 2007, p. 352). This idea, which has been termed as the “self-selection hypothesis” (Trautwein and Lüdtke, 2007), may then explain why students from harder disciplines have higher absolute beliefs regarding scientific knowledge than students from softer disciplines: They might be inclined to choose a hard discipline for their studies (Trautwein and Lüdtke, 2007), and, according to the TIDE framework (Muis et al., 2016; Merk et al., 2018), their general beliefs affect their discipline-specific beliefs. More specifically, the TIDE framework suggests that different levels of epistemic beliefs are reciprocally influential (Muis et al., 2006). Therefore, students with absolute beliefs regarding scientific knowledge in general may not only be attracted by harder disciplines—in fact, their more general absolute beliefs are also likely to deflect on their discipline-specific epistemic beliefs. This, in turn, explains why students from hard disciplines should have higher absolute and lower multiplistic beliefs compared to students from soft disciplines.

While a few articles from the field of personal epistemology refer to Trautwein and Lüdtke’s (2007) self-selection hypothesis (e.g., Eren, 2007; Green and Hood, 2013), it should be noted that much work on its conceptual foundation has yet to be done. Moreover, the empirical evidence regarding the interindividual perspective in general is somewhat mixed. Jehng et al. (1993) found that students with social science and humanities backgrounds viewed knowledge as more uncertain than students from engineering and business. Similarly, Paulsen and Wells (1998) found that students from hard disciplines (e.g., engineering) saw knowledge as more absolute and certain than students from soft disciplines (e.g., social sciences). Both these studies (and several others, see Muis et al., 2006 for an excellent, albeit somewhat dated, literature review), however, employed a domain-general measure, requiring students to give their answers with regard to science “in general.” Hence, since students from hard disciplines likely conceptualize the entity “science” differently (i.e., more absolute) than students from softer fields (Leach et al., 2000), comparability between the groups is reduced and interindividual differences may have been artificially inflated. More recently, using a discipline-specific measure, Karimi (2014) found that English-specific epistemic beliefs differ across individuals, with students from hard disciplines demonstrating higher absolute beliefs compared to students from soft disciplines. Furthermore, again using a discipline-specific measure, Rowley et al. (2008) found that psychology students with and without a biology background espouse largely similar views on psychological knowledge. In contrast, however, they also found that students with a biology background have more absolute beliefs regarding biology than students without a biology background.

With regard to the aforementioned studies, it should be noted that the effect sizes of differences between disciplines are usually smaller under the interindividual compared to the intraindividual perspective, especially in studies using the same discipline-specific instrument across groups (e.g., Rowley et al., 2008). This is not surprising bearing in mind that knowledge structures strongly differ between disciplines (intraindividual perspective), whereas study choices are subject to a multitude of different influences, which likely diminishes self-selection effects (interindividual perspective). Notwithstanding the denoted methodological problems and the small effect sizes, some have argued that students from soft disciplines espouse somehow more “advanced” epistemic beliefs (Paulsen and Wells, 1998; Trautwein and Lüdtke, 2007; Green and Hood, 2013). Based on our deliberations about the self-selection hypothesis, we further investigate this claim regarding the discipline of biology^4^ :

*Hypothesis 2a:* Students from hard disciplines have higher absolute biology-specific beliefs than students from soft disciplines.
*Hypothesis 2b:* Students from hard disciplines have lower multiplistic biology-specific beliefs than students from soft disciplines.

### Combined Perspective: Students From Hard vs. Soft Disciplines and the Perceived Difference Between Psychology and Biology

Self-selection is not the only source of (interindividual) differences in the discipline-specific epistemic beliefs of students from different disciplines. In fact, beliefs evolve and change throughout education (Hofer and Pintrich, 1997; Kuhn et al., 2000). For example, as mentioned above, computer science students develop higher absolute beliefs throughout their studies, and psychology students’ multiplistic beliefs seem to decrease from their second semester on (Peter et al., 2016; Rosman et al., 2017). Such developments are in line with the so-called “socialization hypothesis,” which posits that students’ epistemic beliefs are shaped by the enrollment in specific fields of study (Trautwein and Lüdtke, 2007). In fact, disciplinary contexts may be seen as *socialization agents* (Paulsen and Wells, 1998; Trautwein and Lüdtke, 2007) that influence students’ stance toward scientific knowledge. For example, Trautwein and Lüdtke (2007) argue that soft fields may convey a more nuanced view on the “truth” of scientific theories, and, in their longitudinal study, indeed found that “relative to students enrolled in humanities/arts and (even more so) in the social sciences, participants majoring in engineering and business acquired a less critical epistemological stance over time” (p. 361).

This may be because, in softer disciplines, teaching is usually more student-centered and constructivist. It focuses on critical thinking and on the discussion of multiple points of view (Jones, 2011). Learning how to deal with ambiguity is a central learning goal and knowledge building is seen as a formative process (Paulsen and Wells, 1998; Neumann et al., 2002). In contrast, in harder disciplines, teachers (and curricula) put more focus on teaching basic factual knowledge and on instructing students about how to find “correct” answers (Neumann et al., 2002). According to Paulsen and Wells (1998), such differences in teaching may well deflect on students’ epistemic beliefs, with harder disciplines likely fostering absolute beliefs, whereas studying softer disciplines leads to an endorsement of multiplistic and evaluativistic beliefs. In line with this, Tabak et al. (2010) suggest that “the emphasis on science [makes] broader societal beliefs, such as the belief in the superiority and certainty of science more salient, and that these broader scripts [shape] learners’ views about science” (p. 844).

Furthermore, discipline-specific socialization may also influence the criteria students use to evaluate scientific information. For example, since students from hard disciplines, such as mathematics, seem to be socialized toward finding “correct” answers (Muis et al., 2016), they might perceive disciplines in which finding such answers is more challenging (e.g., psychology) as even more multiplistic and less absolute compared to harder disciplines (Tabak et al., 2010). In contrast, students from soft disciplines are used to the fact that in soft disciplines, evidence is often inconclusive and subject to interpretation (Muis et al., 2006, 2016), making them perceive less of a contrast to harder disciplines. Moreover, due to the stronger focus on critical thinking and empirical research methods in their curricula (Jones, 2011; Muis et al., 2016), they learn how to deal with an inconsistent body of evidence. We therefore expect that they are more likely to recognize that one may approach the “truth” in soft disciplines, which will result, in comparison to students from hard disciplines, in less multiplistic and more absolute views on such soft disciplines. In sum, therefore, we expect students from hard disciplines to perceive a stronger difference between biology and psychology:

*Hypothesis 3a:* The difference between biology- and psychology-specific absolute beliefs is higher for students from hard disciplines than for students from soft disciplines.
*Hypothesis 3b:* The difference between biology- and psychology-specific multiplistic beliefs is higher for students from hard disciplines than for students from soft disciplines.

In addition to these confirmatory hypotheses, we will test whether our expectations regarding Hypothesis 3 are influenced by study duration. In fact, relationships between epistemic beliefs and educational level have long been demonstrated (e.g., Schommer, 1993; King and Kitchener, 2002). Moreover, recent studies suggest that epistemic beliefs also change, in a discipline-specific fashion, over the course of individual study careers, as students learn about characteristics of their discipline, gain knowledge in research methods, and become more competent in evaluating knowledge claims from their discipline. For example, the above-mentioned longitudinal study by Rosman et al. (2017) showed that computer science students’ discipline-specific absolute beliefs decreased over the first few semesters. According to the authors, this is because computer science has a more “absolute” nature, which deflects on the teaching practices, thus conveying “the impression that clear and unambiguous answers are omnipresent in computer science, thus strengthening absolute beliefs” (p. 168). Due to such differences in socialization processes, it might well be that perceived differences between psychology and biology increase, with increasing study duration, in students studying hard disciplines, but decrease in students from soft disciplines. Since this expectation depends on a multitude of (largely untestable) assumptions, we, however, do not formulate confirmatory hypotheses, but label these analyses as exploratory.

## Materials and Methods

### Study Procedure

Hypotheses were tested in a nationwide online study among German university students. Participants were recruited by means of university e-mail distribution, Facebook group announcements, and flyers. As a participation incentive, students could participate, upon study completion, in a lottery of 10*50€ Amazon vouchers.

After an informed consent form and some demographic questions, participants were asked to indicate their study background in free-text and multiple-choice fields (discipline, sought degree, semester). More specifically, they first responded to a series of multiple-choice items asking whether they were currently studying biology, psychology, or teacher training (“Lehramt”). These items were in a forced-choice format and were necessary for the filtering procedure described below. Subsequently, participants were presented with a page on their study background. Students could indicate up to three disciplines in free-text fields, and were, for each discipline, requested to additionally indicate their desired degree (e.g., Bachelor) and their study semester. In order to account for students studying multiple disciplines or degrees in different semesters, study semester data were collected specific for the respective degree (i.e., students were explicitly instructed to respond with a 2 if they studied in their second Master semester). For each free-text field, at least one example was given. Students studying multiple disciplines were asked to indicate all of them. This study background page was presented to all participants except for teacher education students, who were asked about the combination of subjects that their teacher training studies were based on.

In the second part of the test battery, a discipline-specific epistemic beliefs questionnaire (EBI-AM; Peter et al., 2016) was administered three times (see below for details on the measure itself). One version of the questionnaire assessed biology-specific and another one psychology-specific epistemic beliefs, whereas a third version measured students’ epistemic beliefs regarding their own discipline. If a student studied multiple disciplines, the answers in this third version were to be given with regard to the discipline he or she felt most belonging to. To avoid assessing psychology and biology students’ beliefs regarding their own discipline twice, the third version was replaced, for these students, by a version assessing epistemic beliefs pertaining to educational research. Since they study a multitude of subjects, this was also done for teacher education students. Technically, these procedures were realized through a filtering procedure in the survey software (Unipark). To reduce the risk of possible priming effects due to the repeated administration of the questionnaire, the three versions were administered in random order. Hence, even if priming effects would occur due to the repeated administration of the EBI-AM, these would most likely be balanced out across the three versions. Prior to each version, a short description of the discipline in question (psychology, biology, educational research) was given. This was to avoid the risk of participants conceptualizing the respective discipline in different ways (see Estes et al., 2003; Rowley et al., 2008)—which is yet another issue of many of the studies conducted in this field.

### Participants and Classification of Disciplines

Only university students were allowed to participate in the data collection. To reduce invalid participation, a precondition for attending the lottery was an e-mail address from a German university. Of *N* = 1408 participants that had given their consent to participate in the study, *N* = 959 progressed until the end of the questionnaire. With 31.9%, dropout rates were thus as one would expect in online surveys (Galesic, 2006). Doctoral students were discarded from all analyses since they were a rather “exotic’ (and very small) subsample (*n* = 21). The remaining *N* = 938 participants were, on average, *M* = 23.06 (*SD* = 3.17) years old; roughly two thirds of the sample (66.0%) were female. This means that there is a slight over-representation of females in our sample, as the gender distribution at German universities is around 50/50. Participants were studying a multitude of degrees (Bachelor, Master, Diploma, etc.) and were enrolled in a broad range of semesters.

To classify participants’ study disciplines, a multi-step approach was adopted. First, based on an initial screening of the disciplines included in the dataset, a classification scheme was developed by the two first authors of this paper (see Table 1). The scheme consisted of 22 broad disciplinary clusters such as “education,” “physics,” or “mathematics.” It was based on existing classifications (e.g., Biglan, 1973; Stoecker, 1993; Simpson, 2017; DESTATIS, 2018), but, due to the heterogeneity of disciplines within our dataset, some classifications had to be adapted. The scheme was realized as a spreadsheet including several examples for each disciplinary cluster, and also included a set of rules on how to classify exceptional cases [e.g., for disciplines with a combined name (“food chemistry”), only the last name (“chemistry”) was to be regarded]. In a second step, the 22 disciplinary clusters were classified as either hard or soft (see Table 1) according to the classification of the Biglan scheme (Biglan, 1973; Stoecker, 1993; Simpson, 2017). In a third step, two independent student assistants categorized all disciplines according to the new classification scheme, with the exception of psychology, biology, and teacher education, where the corresponding data had already been collected through multiple-choice questions in the survey software (which were required for the filtering procedure described in section “Study Procedure”). Both student assistants were blind to our hypotheses. Interrater reliability between the two coders was excellent with a Cohen’s Kappa of κ = 0.98. In cases where both raters disagreed, the two first authors of this manuscript agreed on one classification. The number of students in different disciplines can be found in Table 1. Approximately one-third of the sample (*n* = 348, 37.6%) was classified as belonging to a hard discipline, which, as a side note, approximates the actual distribution of STEM students in Germany quite well (38.4% in 2016/2017; DESTATIS, 2019a). It should be noted that the gender distribution was not equal across disciplines—in fact, the proportion of males was significantly higher (χ^2^ = 73.32; *p* < 0.001) in hard disciplines compared to soft disciplines. This, however, is also in line with the actual distributions in Germany (DESTATIS, 2019b).

**TABLE 1:** Classification of disciplines.

**Number of cases**	**Disciplinary cluster**	**Examples**	**Biglan classification**
78	Biology	Biology (yes/no item), Molecular Biosciences, Neurosciences	Hard
14	Chemistry	Chemistry, Food Chemistry	Hard
81	Computer Science	Computer Science, Geoinformatics, Business Informatics	Hard
37	Economics	Business Administration, Political Economics, Marketing	Soft
47	Education	Educational Research, Social Pedagogy, Adult Education	Soft
50	Engineering/Technology	Mechanical Engineering, Electrical Engineering, Biomedical Technology	Hard
25	Geography	Geography, Meteorology, Environmental Geography	Hard
19	History	History, Art History, Archeology	Soft
24	Languages and Literature	Japanology, English Literature, German Language Studies	Soft
44	Law	Law, Jurisprudence	Soft
19	Mathematics	Mathematics, Business Mathematics	Hard
18	Media and Communication	Media Science, Media and Communication	Soft
57	Medicine	Medicine, Dental Medicine	Hard
2	Philosophy	Philosophy	Soft
21	Physics	Physics	Hard
12	Political Science	Political Science, Political and Social Studies	Soft
140	Psychology	Psychology (yes/no item)	Soft
25	Sociology	Sociology, Sociology of Culture	Soft
202	Teacher Education	Teacher Education (yes/no item)	Soft
2	Religion	Interreligious Studies, Islamic Studies	Soft
21	(Unclassified)	Architecture, Digital Humanities, Environmental Science, Ethnology, Forest Science, Nursing, Social Science	Soft (except Environmental Science)

### Measures

As already mentioned above, epistemic beliefs were assessed using the EBI-AM questionnaire by Peter et al. (2016). We chose this questionnaire since it allows assessing absolutism (12 items) and multiplism (11 items) on separate scales, thus being more closely related to our theoretical framework (i.e., Kuhn and Weinstock, 2002) than other questionnaires. A second reason for choosing the EBI-AM is that both absolutism and multiplism are assessed with regard to the four dimensions from the 1997 framework by Hofer and Pintrich (e.g., both the absolutism and multiplism scales contained items pertaining to certainty, simplicity, source, justification of knowledge; 5–7 items per dimension; see Peter et al., 2016). Even though these dimensions’ construct validity has not been tested yet, their presence points to the broad scope of the inventory, which should thus allow a comprehensive assessment of epistemic beliefs. Finally, the EBI-AM’s design allows an easy adaptation to different disciplines—we simply changed the questionnaire’s introduction to include the different disciplines we were interested in (e.g., “Please relate your answers to the scientific field of [psychology/biology]”). Moreover, in the items themselves, we replaced the expression “in this discipline” (see Peter et al., 2016) by the respective discipline of interest. For example, high scores on items such as “There is always a true answer to questions in biology” indicates high biology-specific absolutism; high scores on the item “In psychology, only uncertainty appears to be certain” indicates high psychology-specific multiplism. All items were answered on a five-point Likert scale ranging from “do not agree at all” to “fully agree.” Scores were obtained by calculating mean scores across all items of one scale (e.g., absolutism). Factor structures were investigated as has been done before (e.g., Merk et al., 2017), using a CFA model (τ-generic measurement models) with two first-order factors indicating the developmental stage (absolutism and multiplism), and four first-order factors indicating the dimensions by Hofer and Pintrich (1997) resulting in cross-loadings for every item. For the psychology-specific EBI-AM, model fit was good (CFI = 0.937; TLI = 0.921; RMSEA = 0.037; SRMR = 0.034) and scale reliabilities, which were estimated using McDonald’s ω, were acceptable (absolutism: ω = 0.78; multiplism: ω = 0.75). Similarly, the biology-specific EBI-AM yielded good CFA fits (CFI = 0.957; TLI = 0.946; RMSEA = 0.037; SRMR = 0.031) and rather high reliability estimates (absolutism: ω = 0.86; multiplism: ω = 0.81).

Study duration was assessed based on the study semester data collected on the study background page (see above). The study semester variable, however, had to be recoded since, as outlined above, study semester had been measured in a way that was specific for the respective degree. Therefore, in the raw study semester data, a person studying in her second Master semester has a score of 2, and someone studying in his fourth Bachelor semester has a score of 4—which obviously does not correctly reflect study duration. To account for this, the study duration score was increased by 6 for all Master students (Bachelor studies usually take around six semesters in Germany). It should be noted that our study duration variable thus reflects an approximation of the actual study duration since some students might, for example, have taken longer for their Bachelor studies—yet another reason to only conduct exploratory analyses with regard to study duration.

### Data Analysis Preparation

As predicted by the socialization hypothesis (Trautwein and Lüdtke, 2007), studying a specific discipline entails socialization effects that may well affect discipline-specific epistemic beliefs. Therefore, biology students might conceptualize “biology” in a fundamentally different way compared to students not studying biology. Since this might introduce bias in our analyses and since we were interested in the *general* population of university students (and not in the populations of specific disciplines or in students’ perception of their *own* discipline), biology and psychology students were excluded from all analyses that included biology- respectively psychology-specific epistemic beliefs. More specifically, biology students were excluded from all of our analyses (since all hypotheses included biology-specific epistemic beliefs), and psychology students were excluded from all analyses regarding hypotheses 1 and 3 (since only these hypotheses included psychology-specific epistemic beliefs). In addition, since the present article focuses on comparisons between biology and psychology and since the ratings on students’ own disciplines are hard to interpret due to the multitude of different disciplines in our sample, data on students’ beliefs regarding their own discipline were not analyzed.

All study hypotheses were tested using Bayes factors^5^ with JZS priors (Rouder et al., 2012). We chose this approach to circumvent (at least some) of the problems associated with *p* values (Wagenmakers et al., 2018): As the Bayes factor (BF_10_) is defined as the proportion of the marginal likelihoods of two competing Models ℳ_1_ and ℳ_0_

$$B\,F_{10}=\frac{p\,\left(D|{ℳ}_{1}\right)}{p\,\left(D|{ℳ}_{0}\right)}$$

it can tell us that the data are more likely to be observed under ℳ_1_ (if BF_10_ > 1) as well as the opposite. Given the research questions at hand, this is quite useful, as we can gain evidence for the hypothesis of different means in two groups (specificity of beliefs) as well as for the hypothesis of two equal means (generality of beliefs). In other words, we cannot only gather evidence for the alternative hypothesis that there is a difference between two groups or two variables, but also for the null hypothesis (which suggests that there is no difference). In particular, the latter is not possible with classical *p* values (Wagenmakers et al., 2018)—interpreting a “non-significant” result (i.e., with a *p*-value over 0.05) as suggesting that there is no difference between two means is not correct (Dienes, 2014). Moreover, a *p*-value *under* 0.05 would support the rejection of the null hypothesis, but would, strictly speaking, not allow to make inferences regarding the acceptance of the alternative hypothesis (Wagenmakers et al., 2018). All analyses were conducted in R using the BayesFactor 0.9.12-4.2. package (Morey et al., 2018).

### Compliance With Ethical Standards

The study is in full accordance with the Declaration of Helsinki and the APA Ethics Code (American Psychological Association, 2002). Prior to their participation, students received an informed consent form including, among others, (1) a statement on the purpose of the research as well as the expected study duration and procedures; (2) a statement that participation is voluntarily and that it may be terminated at any point; (3) a statement that there are no potential risks, discomfort, or adverse effects with regard to their participation; and (4) a statement that data are collected anonymously. Explicit agreement to the informed consent form was required at the beginning of the study.

## Results

All analyses were conducted using factor scores. As shown in Figures 2–4, some of our data were skewed, which is why we computed Vargha and Delaney’s *A*_12_ (Vargha and Delaney, 2000) to judge the magnitude of the mean differences. *A*_12_ is also called a measure of stochastic superiority. This means that a value of *A*_12_ = 0.60 can be interpreted as the probability that a randomly drawn data point from distribution 1 is greater than one from distribution 2. Hence *A*_12_ = 0.50 means that there is no effect, whereas *A* values close to 0 and 1 imply very large effects.

### Hypothesis 1

**FIGURE 1:**
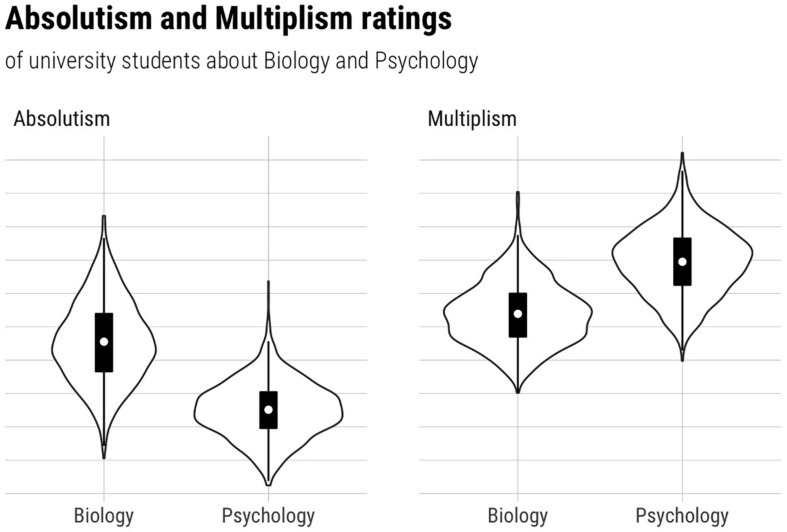
Differences between biology-specific and psychology-specific epistemic beliefs (intraindividual perspective).

Hypothesis 1 aimed at testing differences between biology-specific and psychology-specific epistemic beliefs of university students in general. We expected that university students would have higher biology-specific absolute beliefs compared to psychology-specific absolute beliefs (H1a), and that they would have lower biology-specific multiplistic beliefs compared to psychology-specific multiplistic beliefs (H1b). Descriptively (see Figure 1 and Table 2), both effects are in the expected direction, exhibiting very large effect sizes (Vargha and Delaney, 2000) for both H1a (*A* = 0.088) and H1b (*A* = 0.874) since both *A* values clearly differ from 0.50 (see above). For H1a, a Bayesian *t-*test for paired samples based on JZS priors with *r* = [QSIImage] resulted in Bayes factor of *BF* = 1.262^∗^e^211^, meaning that the data at hand are more than 1,000 times more likely under the assumption of a mean difference between biology specific absolutism and psychology specific absolutism than under the assumption of equal means. Similarly, for H1b, a Bayes factor of *BF* = 1.524^∗^e^231^ indicates that the data at hand are more than 1,000 times more likely under the assumption of a mean difference compared to the assumption of equal means. In sum, this indicates that there is very strong evidence (Jeffreys, 1998) for H1a and H1b, combined with very large effect sizes.

**TABLE 2:** Intraindividual differences in psychology- and biology-specific epistemic beliefs.

		***M***	***SD***	**1**	**2**	**3**	**4**
1	Psychology-specific absolute beliefs	2.34	0.52	**(0.78)**			
2	Biology-specific absolute beliefs	2.95	0.69	0.42***	**(0.75)**		
3	Psychology-specific multiplistic beliefs	3.33	0.52	−0.29***	–0.03	**(0.86)**	
4	Biology-specific multiplistic beliefs	2.74	0.58	−0.09*	−0.54***	0.36***	**(0**.**81)**

N = 718–719 (psychology and biology students omitted); M, arithmetic mean; SD, standard deviation; values in bold on the diagonal = McDonald’s ω; *p < 0.05; ***p < 0.001 (two-tailed tests).

### Hypothesis 2

**FIGURE 2:**
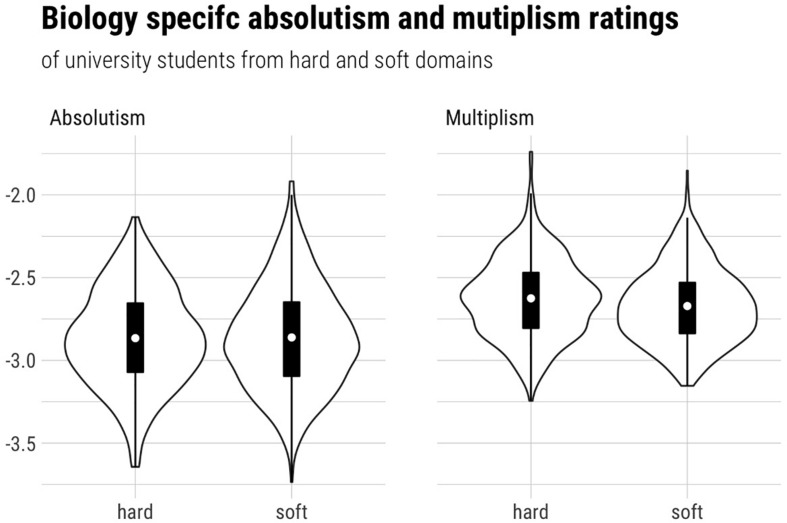
Differences between students from hard and soft disciplines in biology-specific epistemic beliefs (interindividual perspective).

Hypothesis 2 aimed at testing differences between students from hard and soft disciplines. We expected that students from hard disciplines have higher absolute (H2a) and lower multiplistic (H2b) biology-specific epistemic beliefs. However, on a descriptive level (see Figure 2 and Table 3), there were almost no mean differences regarding absolutism, which was corroborated by the effect size calculations indicating a negligible effect (*A* = 0.513). Bayesian *t*-tests (for independent samples, JZS priors with *r* = [QSIImage]) revealed a Bayes factor of *BF* = 0.084, which can be seen, according to Jeffreys (1998), as substantial evidence for the null hypothesis that students from hard and soft domains do not differ in their average ratings of biology-specific absolutism (in comparison to the hypothesis of unequal means). Concerning H2b, descriptive analyses showed that multiplism was, opposite to our expectations, slightly higher for students studying hard disciplines. Effect size calculations revealed a small corresponding effect (*A* = 0.574), and our Bayesian analyses yielded a Bayes factor of *BF* = 25.706. Hence, with regard to common rules of thumb (e.g., Jeffreys, 1998), our data provide strong evidence that students from hard and soft domains do differ in their average ratings of biology-specific multiplism—but in the opposite direction of what we had expected.

**TABLE 3:** Interindividual differences between students from hard and soft disciplines in biology-specific epistemic beliefs.

	**Hard**			**Soft**		
	**disciplines**			**disciplines**		
	***n***	***M***	***SD***	***n***	***M***	***SD***
Biology-specific absolute beliefs	270	2.98	0.65	577	2.91	0.69
Biology-specific multiplistic beliefs	270	2.75	0.58	577	2.69	0.56

*Biology students and unclassified cases omitted; *n*, subgroup sample size; *M*, arithmetic mean; *SD*, standard deviation.*

Since these results were somewhat unexpected, we conducted an additional exploratory analysis of Hypothesis 2 with regard to psychology-specific beliefs (instead of biology-specific beliefs), which however revealed a very similar pattern of results (no differences regarding psychology-specific absolutism, but higher psychology-specific multiplism in students from hard sciences).

### Hypothesis 3

**FIGURE 3:**
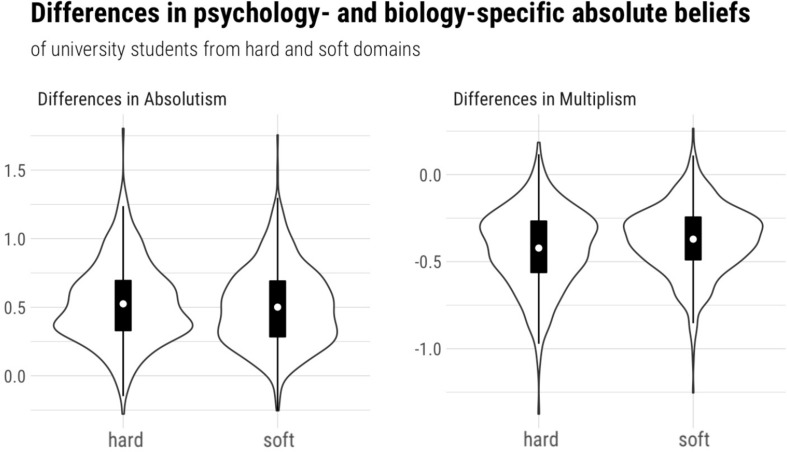
Differences between biology- and psychology-specific epistemic beliefs in relation to participants’ field of studies (combined perspective).

Hypothesis 3 combined the intraindividual with the interindividual perspective by suggesting that the difference between biology- and psychology-specific absolute (H3a) and multiplistic beliefs (H3b) would be higher for students from hard disciplines than for students from soft disciplines. To illustrate this hypothesis graphically, two difference scores were calculated and plotted in Figure 3 (“Differences in absolutism” = biology-specific absolutism - psychology-specific absolutism; “Differences in multiplism” = biology-specific multiplism - psychology-specific multiplism). Positive values on this score indicate that biology is perceived as more absolute (or more multiplistic) than psychology; negative values indicate the opposite. A descriptive glance at the data reveals, with regard to absolutism, support for H3a since the difference between biology- and psychology-specific absolute beliefs was indeed higher for students from hard disciplines (see Table 4 and Figure 3; difference scores are for illustrative purposes only). To test Hypothesis 3, we estimated Bayes factors for mixed ANOVA designs (Rouder et al., 2012), which drew on a comparison between a model including the main effects of the within factor (discipline) and the between factor (hard/soft) and a model including both main effects and their interaction. This yielded a Bayes factor of *BF* = 1.156 for absolutism. Hence, the data are more or less equally likely under both models. Concerning multiplism, however, the difference between psychology and biology descriptively seems a bit larger for students from hard disciplines—which is in line with the predictions of H3b. An analogous Bayes factor analysis to the one above resulted in a *BF* = 9.158, which suggests that the data at hand are 9.158 times more probable under the assumption of the more complex model (including the interaction between within and between factor) compared to the assumption that there are only main effects. However, it should be noted that this is only a very small interaction effect ([QSIImage] = 0.003). In sum, our calculations thus provide evidence for H3b, but not for H3a.

**TABLE 4:** Inter- and intraindividual differences between students from hard and soft disciplines in biology- and psychology-specific epistemic beliefs.

	**Hard**			**Soft**		
	**disciplines**			**disciplines**		
	***n***	***M***	***SD***	***n***	***M***	***SD***
Biology-specific absolute beliefs	270	2.98	0.65	437	2.93	0.71
Psychology-specific absolute beliefs	270	2.30	0.48	436	2.36	0.54
Biology-specific multiplistic beliefs	270	2.75	0.58	437	2.73	0.57
Psychology-specific multiplistic beliefs	270	3.41	0.55	436	3.28	0.50

*Psychology students, biology students, and unclassified cases omitted; *n*, subgroup sample size; *M*, arithmetic mean; *SD*, standard deviation.*

**FIGURE 4:**
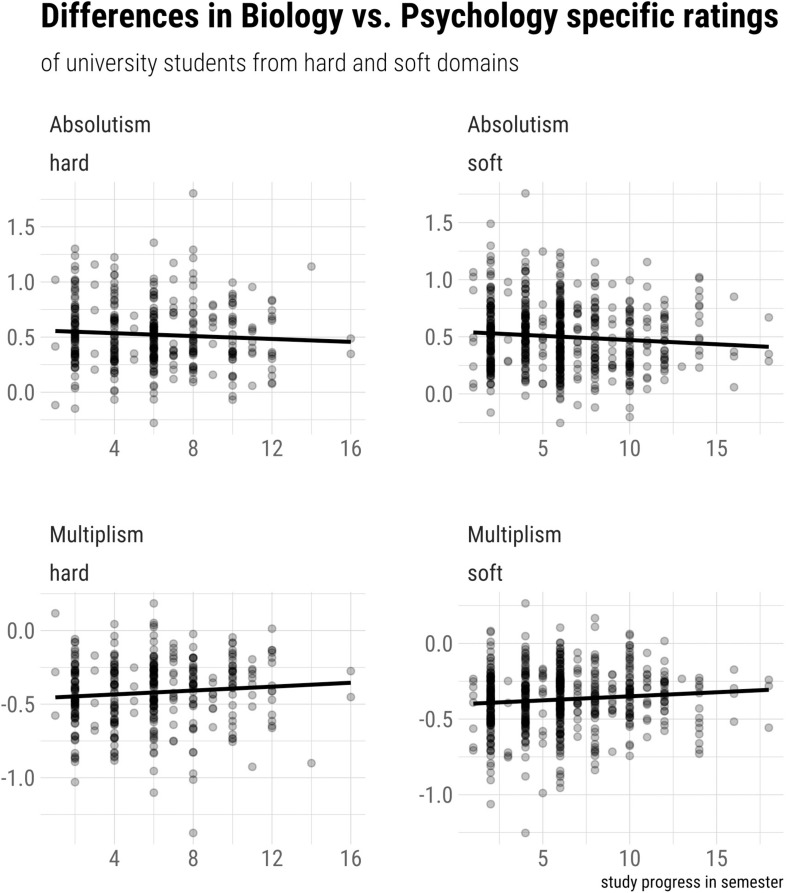
Differences between biology- and psychology-specific epistemic beliefs in relation to participants’ study duration and field of studies.

To investigate whether these results may be influenced by study duration, we conducted two additional exploratory Bayesian analyses (one for absolutism and one for multiplism). In these analyses, we compared a linear model containing study duration and the hard/soft variable against a model containing only the hard/soft variable. To increase the interpretability of these rather elaborate models, the respective dependent variables were operationalized as difference scores (see above), hence allowing us to omit the within factor as a predictor variable. Regarding absolutism, we found moderate evidence (*BF* = 0.105) that controlling for study duration does *not* impact our results. In other words, regarding absolutism, there seems to be no difference between students from hard and soft differences in the relationship between study duration and the perceived difference between psychology and biology. Similarly, regarding multiplism, the corresponding Bayesian analyses yielded moderate, albeit somewhat weaker (*BF* = 0.319) evidence that controlling study for duration does not impact our results. These results are illustrated in Figure 4.

## Discussion

The present research investigated whether university students’ discipline-specific epistemic beliefs vary with regard to different disciplines, and whether this variation depends on the students’ own field of studies. To test our predictions, we conducted a nationwide online study among German university students, who indicated their discipline and responded to psychology- and biology-specific epistemic beliefs questionnaires. To allow a meaningful interpretation of “non-significant” findings, data were analyzed via Bayesian statistics (Wagenmakers et al., 2018).

### Hypothesis 1

Our findings regarding Hypothesis 1 reveal substantial intraindividual differences in how university students perceive psychology and biology. Of note is that this hypothesis had a rather firm empirical basis, even before our data collection (i.e., several prior studies with similar results, see section “Intraindividual Perspective: Psychology- vs. Biology-Specific Epistemic Beliefs”). This, together with the strong evidence our analyses provided, further strengthens our belief that biology is seen as considerably more absolute and less multiplistic than psychology. Among others, this is in line with the findings by Muis et al. (2016), who found that psychological knowledge is seen as more multiplistic—at least compared to mathematical knowledge. Moreover, our pattern of results confirms the findings by Estes et al. (2003) and Rowley et al. (2008), who found biological knowledge to be perceived as more certain and trustworthy compared to psychological knowledge. This suggests that differences in how a discipline is taught and presented in the educational context (e.g., in university courses) may well affect epistemic beliefs (Rowley et al., 2008). Furthermore, on a conceptual level, our findings serve as further evidence for the discipline specificity of epistemic beliefs and for the usefulness of measuring epistemic beliefs at the discipline level. Therefore, our findings also support a key assumption of the TIDE framework, which is literally built around the idea of different levels of specificity within an individual’s belief system (Muis et al., 2006). In this regard, the convergence between the Biglan scheme’s predictions (biology as hard, psychology as soft; e.g., Simpson, 2017) and our results (biology as more absolute than psychology, psychology as more multiplistic than biology) is also noteworthy. In fact, the Biglan classification scheme has been validated in several studies not only involving sorting tasks and surveying of faculty members (Biglan, 1973; Smart and Elton, 1982; Stoecker, 1993), but also more “objective” methods such as Simpson’s (2017) correspondence analysis of the distribution of disciplines across universities. The correspondence between our predictions and the Biglan scheme thus supports the idea that discipline-specific epistemic beliefs reflect the nature and structure of discipline-specific knowledge. An alternative explanation, however, would be that our findings are caused by stereotypes and misconceptions that university students hold regarding knowledge in biology and/or psychology. Since our data do not allow corresponding analyses (we would need a “reference group” such as, e.g., faculty members), future research should analyze to what extent stereotypes shape students’ epistemic beliefs.

### Hypothesis 2

Hypothesis 2 predicted that students from hard disciplines would perceive biology as more absolute and less multiplistic compared to students from soft disciplines. However, as outlined above, this hypothesis had a less strong empirical foundation compared to Hypothesis 1. For example, Rowley et al. (2008) found that students with and without a biology background had quite similar views on psychological knowledge, and other studies that found the denoted differences exhibit methodological problems (see section “Interindividual Perspective: Differences in Epistemic Beliefs Between Students From 149 Hard and Soft Disciplines”). Our Bayesian analyses, in turn, yielded substantial evidence for the *null* hypothesis with regard to biology-specific absolute beliefs—that is, that there would be *no* differences between students from hard and soft disciplines. Moreover, our analyses provided evidence for multiplism being slightly higher in students studying hard disciplines, which is opposite to our predictions. Several explanations for these unexpected findings come to mind. First, the self-selection hypothesis (Trautwein and Lüdtke, 2007), which had driven our expectation that students would choose a discipline that is aligned with their prior beliefs, might be less strong than expected. A second explanation is that the self-selection hypothesis might well hold, namely, that students with higher general absolute beliefs are inclined to choose more “absolute” disciplines, but that these general beliefs do not permeate to discipline-specific epistemic beliefs. This would speak against the TIDE framework’s prediction that different levels of epistemic beliefs are reciprocally influential (Muis et al., 2006). It should, however, be noted that our study design does not allow delineation between these two explanations, which is why further research should investigate the issue. In sum, our results concerning Hypothesis 2 thus support Rowley et al. (2008) idea that “epistemological thinking is shaped by domain-related experience, rather than some underlying aspect of disposition” (p. 23). This alternative possibility, the shaping of epistemic beliefs by domain-related experience, was investigated in Hypothesis 3.

### Hypothesis 3

Our findings regarding Hypothesis 3 revealed that the difference between psychology- and biology-specific multiplistic beliefs is perceived as slightly stronger by students from hard disciplines compared to those enrolled in soft disciplines. This provides evidence for the socialization hypothesis, which suggests that students’ epistemic beliefs are shaped by the enrolment in specific fields of study (Trautwein and Lüdtke, 2007): Students from hard disciplines might have been socialized toward preferring “hard” evidence, which would imply a devaluation of soft disciplines such as psychology. We, however, concede that this interpretation is somewhat speculative, especially since, in our exploratory analyses, we did not find that the findings regarding Hypothesis 3 depend on study duration: According to the socialization hypothesis (Trautwein and Lüdtke, 2007), the difference between psychology and biology would increase with study duration—but only in students from hard disciplines. Students from soft disciplines, in contrast, might learn how to deal with ill-defined evidence during their studies (e.g., in research methods courses), and might therefore perceive smaller differences between hard and soft disciplines over time. Since we did not find a corresponding interaction with study duration, our evidence regarding the socialization hypothesis is, in our view, rather limited. However, it should be noted that the longitudinal design of Trautwein and Lüdtke’s (2007) study is better suited to investigate socialization effects compared to our cross-sectional approach. Therefore, to clarify the issue, future research should adopt a longitudinal perspective. Moreover, it should be pointed out that Hypothesis 3 was supported only with regard to multiplism (i.e., H3b), but our results remained inconclusive with regard to absolutism (H3a). Hence, with a Bayes factor of around 1, we can neither confirm nor reject H3a, which is why we refrain from an interpretation of this result. What seems clear, however, is that all effect sizes regarding Hypothesis 3 were rather small (possibly also explaining the absence of effects regarding absolutism), and, overall, the evidence was less strong than regarding Hypothesis 1.

### Limitations and Future Directions

A main advantage of our study is its large and heterogeneous sample as well as the elaborate data analysis procedure. Other studies investigating similar research questions often rely on students from a fixed set of disciplines (e.g., Rowley et al., 2008; Karimi, 2014), thus reducing generalizability to other samples. Along with this heterogeneity, however, comes a downside, because classifying disciplines into a fixed number of categories (the basis of our hard/soft classification) is always somewhat arbitrary. Moreover, our analyses focus on biology- and psychology-specific epistemic beliefs only. While we agree that this selection of disciplines is also arbitrary, we emphasize that analyzing the beliefs on multiple disciplines would bear several new issues, such as fatigue effects due to the repeated administration of virtually the same measure. In fact, even administering the EBI-AM three times, as we did in our study, may have led to a certain amount fatigue or priming effects, thus decreasing effect sizes. It should also be noted that we did not consider the two other dimensions of Biglan’s (1973) classification (i.e., pure/applied and life/non-life). This was for reasons of statistical power and interpretability of our results. We nevertheless point out that further distinctions in Biglan’s classification might nuance our findings, especially since Donald (1990) found that professors from pure and applied disciplines differed in their validation processes and truth criteria used to evaluate evidence.

What may be perceived as another limitation is that we only assessed epistemic beliefs at one level of specificity. When testing their extension of the TIDE framework, Merk et al. (2018) however found that epistemic beliefs also vary over different topics within a specific discipline, and Muis and Gierus (2013) found that individuals’ beliefs might vary within a domain depending on the specific context within a domain. Therefore, taking an even more fine-grained perspective might also prove worthwhile in future research. Related to this, one might also increase the scope of our findings by adopting a person-centered approach (e.g., Kampa et al., 2016) and investigating our research questions at the level of different student groups, for example, using latent profile analysis.

Another limitation is our sole use of self-report measures. In fact, while the EBI-AM performed rather well in our study, psychometric issues (reliability problems and failures to replicate factor structures) are common in the measurement of epistemic beliefs (Greene and Yu, 2014; Mason, 2016). Moreover, validity concerns have been raised since self-reports may not capture all aspects of the complex construct in question (Mason, 2016). In our opinion, such issues play an even more important role in belief types that are harder to verbalize, such as evaluativism. As outlined earlier, we therefore did not include an evaluativism measure, which doubtlessly constitutes another limitation of our study. The latter two issues might be tackled by scenario-based assessments that include evaluativism (e.g., Barzilai and Weinstock, 2015; Rosman et al., 2019; Iordanou et al., 2019), or through qualitative interviews and analyses of trace data (e.g., Greene and Yu, 2014). Such instruments, however, are not easily adapted for different disciplines, which is why we chose an established and reliable discipline-specific self-report instrument. Nevertheless, future research should strive to investigate corresponding research questions with regard to evaluativism. For example, one might expect that, due to differences in knowledge structures (e.g., Jones, 2011; Muis et al., 2016), students perceive softer disciplines as requiring more weighing of different viewpoints compared to harder disciplines. Moreover, with regard to interindividual differences, students from softer disciplines might, in line with the socialization hypothesis (Trautwein and Lüdtke, 2007) report higher evaluativistic beliefs since they are socialized toward weighing and evaluating evidence.

## Conclusion

In conclusion, our findings show that there is a considerable amount of intraindividual variance in university students’ epistemic beliefs, at least when contrasting psychology and biology. On an interindividual level, however, our analyses suggest that there are no strong differences (if any at all) between students from hard and soft disciplines regarding the perception of biology. This disconfirms earlier interpretations that soft fields might “produce” somehow different epistemic beliefs, as is exemplified by Green and Hood arguing that students “from ‘soft’ and ‘pure’ disciplines [have] significantly more sophisticated [epistemic beliefs] than … students from ‘hard’ or ‘applied’ disciplines” (Green and Hood, 2013, p. 170). While our analyses also revealed that students enrolled in harder disciplines perceive a stronger difference between psychology and biology, these effects were rather small and our expectations were only supported on one dependent variable (multiplism). In sum, our study, with its large sample and its elaborate analysis procedure, nevertheless provides some compelling evidence that, as we hope, will contribute to a nuanced discussion on whether students from soft disciplines espouse somehow “better” or “different” beliefs than their peers from hard disciplines. Considering the knowledge gained throughout our analyses, we, for our part, suggest that the devaluation and denigration of science, as indicated by high multiplism and low epistemic trust, may happen in any discipline. We therefore think that developing interventions that are suited for a broad range of students should be a key element of future research in the growing field of epistemic cognition.

## Data Availability Statement

The datasets generated for this study are available on request to the corresponding author.

## Ethics Statement

Ethical review and approval was not required for the study on human participants in accordance with the local legislation and institutional requirements. Written informed consent from the participants’ legal guardian/next of kin was not required to participate in this study in accordance with the national legislation and the institutional requirements.

## Author Contributions

TR and ES conceived, planned, and conducted the experiment. TR and ES prepared the data analysis and also conducted a first basic data analysis. SM conducted the Bayesian data analysis and wrote a first draft of the results section. TR prepared the first draft of the other sections. Subsequently, all authors critically reviewed and revised the manuscript.

## Conflict of Interest

The authors declare that the research was conducted in the absence of any commercial or financial relationships that could be construed as a potential conflict of interest.

## Footnotes

1 The term “domain-specific” is, in our opinion, a bit vague since it does not delineate between the level of more general academic beliefs (i.e., beliefs regarding the academic “domain”) and the level of specific disciplines (e.g., beliefs regarding the mathematics “domain”). From now on, we will use the more precise term “discipline-specific” instead of “domain-specific.”

2 It should be noted that we do not intend to state that discipline-specific epistemic beliefs *only* reflect the knowledge structure of a specific discipline. As will become evident in later sections of the present article, other aspects such as socialization are undoubtedly important, too. Nevertheless, we think that the role of discipline-specific knowledge structures should not be underestimated, especially in research on within-person differences.

3 It should be noted that no hypotheses regarding evaluativism, the third stage of Kuhn and Weinstock’s (2002) model, are specified. This is because we are not aware of any existing instruments suited to reliably assess evaluativism that are adaptable for different disciplines. In fact, measuring evaluativism using multiple choice items often produces ceiling effects, partially due to social desirability and due to the fact that evaluativistic items (e.g., “Ideas in science sometimes change”; Conley et al., 2004, p. 203) are hard to disentangle from multiplistic items (e.g., “Ideas in science change all the time”; see also Peter et al., 2016). A qualitative assessment of evaluativism (e.g., by means of interviews; Greene and Yu, 2014) circumvents the issue, but is, obviously, not feasible in a large-scale online study. Another promising alternative would be scenario-based assessments (e.g., participants read a scenario depicting some scientific controversy, followed by Likert-scale items assessing their thoughts on the controversy; Barzilai and Weinstock, 2015; Rosman et al., 2019). However, such scenario-based assessments are usually topic-specific (i.e., they measure students’ beliefs regarding the topic specified in the scenario) and are very hard to transfer to the context of an entire discipline. For these reasons, we did not include hypotheses regarding evaluativism.

4 For reasons of parsimony, we did not formulate confirmatory hypotheses with regard to psychology-specific beliefs (but see the exploratory analysis in section “Hypothesis 2” of Results section).

5 Prior to the Bayesian analyses, we also analyzed our data with more ‘traditional’ methods (e.g., *t*-tests, analyses of variance), which yielded largely similar results.
